# Exosomal miRNA‐21‐5p and miRNA‐21‐3p as key biomarkers of myocardial infarction

**DOI:** 10.1002/hsr2.2228

**Published:** 2024-07-09

**Authors:** Reza Sahebi, Fatemeh Gandomi, Mitra shojaei, Effat Farrokhi

**Affiliations:** ^1^ Department of Molecular Medicine, School of Advanced Technologies Shahrekord University of Medical Sciences Shahrekord Iran; ^2^ Metabolic Syndrome Research Center, School of Medicine Mashhad University of Medical Sciences Mashhad Iran

**Keywords:** exosome, extracellular vesicles, miRNA‐21, myocardial infarction, ROC curve

## Abstract

**Objective:**

Coronary artery disease (CAD) is a debilitating condition that can lead to myocardial infarction (MI). Exosomal miRNAs (exo‐miRNA) can be diagnostic biomarkers for detecting MI. Here, we conduct a study to evaluate the efficacy of exo‐miRNA‐21‐5p/3p for early detection of MI.

**Methods:**

A total of 135 CAD patients and 150 healthy subjects participated in this study. Additionally, we randomly divided 26 male Wistar rats (12 weeks old) into two groups: control and induced MI. Angiographic images were used to identify patients and healthy individuals of all genders. In the following, serum exosomes were obtained, and exo‐miRNA‐21‐5p/3p was measured by reverse‐transcriptase polymerase chain reaction.

**Results:**

We observed an upregulation of exo‐miRNA‐21‐5p/3p in CAD patient and MI‐induced animal groups compared to controls. Analysis of the ROC curves defined 82% and 88% of the participants' exo‐miRNA‐21‐5p and exo‐miRNA‐21‐3p diagnostic power, respectively, which in the animal model was 92 and 82.

**Conclusion:**

This study revealed that the mean expression levels of exo‐miRNA‐21‐5p/3p were significantly increased in CAD patients and animal models of induced MI. Also, these results are associated with the atherogenic lipid profile of CAD patients, which may play an important role in the progression of the disease. Therefore, they can be considered as novel biomarkers.

## 1 INTRODUCTION

Coronary artery disease (CAD) occurs when coronary arteries narrow due to atherosclerosis or clots, causing insufficient oxygen supply, angina, and myocardial infarction (MI).^1^, ^2^ A proper diagnostic technique for the detection of CAD patients will help physicians initiate treatment more efficiently.^3^ Diagnosis, follow‐up, and treatment of CAD involve numerous diagnostic methods, but common tests like exercise electrocardiography,^4^ coronary angiography,^5^ and myocardial perfusion single‐photon emission computed tomography (SPECT) are limited by low sensitivity, invasiveness, high cost, accessibility, and radiation exposure.^6^, ^7^ Although traditional methods like serum levels of troponin (Tn) and creatine kinase‐MB (CK‐MB) can diagnose MI, these enzymes typically increase after cardiomyocyte apoptosis and MI.^8^ Therefore, the existence of a novel diagnostic target for early detection of heart cell damage is crucial.^9^ Extracellular vesicles (EV) or exosomes, with an average diameter of 30–100 nm, could be a new diagnostic method, containing genetic information like DNA, mRNA, and microRNA (miRNAs) that can transmit various signals between different cells.^10^ MiRNAs are small noncoding RNAs with several activities, such as cell proliferation, gene regulation, and pathophysiological progression.^11^, ^12^ Exosomal miRNAs (exo‐miRNAs), which are more stable than plasma miRNAs due to their protection from body fluid degradation,^13^ have been linked to heart injuries in humans and animals.^14^ Tao Geng et al. showed exo‐miRNA‐143 decreased in MI, which enhanced angiogenesis.^15^ Evidence indicates that some miRNAs significantly contribute to the progression of heart disease, including myocardial ischemia, while others enhance function.^16^, ^17^ Impaired blood flow in CAD can alter miRNA expression, leading scientists to study miRNA‐21 as a potential diagnostic biomarker due to its significant difference in expression in CAD patients compared to healthy subjects.^18^ The gene of miRNA‐21, located on chromosome 17q23.2, is responsible for the precursor miRNA‐21's transcription and processing. This process creates two mature strands: the guide strand (miRNA‐21‐5p) and the passenger strand (miRNA‐21‐3p), which degrade due to lower stability.^19^ Mengwen Yan et al. revealed that miRNA‐21‐3p expression increases in heart failure patients and can control cardiac hypertrophic by inhibiting HDACs, which activate the Akt/Gsk3b pathway.^20^ Consistent with Mengwen Yan, WeiHuang showed that miRNA‐21‐5p inhibits cardiomyocyte apoptosis by restricting the PTEN/AKT pathway. In addition, Argonaute‐miRNA‐21 (agomiRNA‐21) increased ejection fraction (EF) status and reduced the size of the infarct area in mice.^21^ Therefore, many studies have emphasized the therapeutic and diagnostic effects of miRNA‐21. Zhou et al. suggest that miRNA‐21 may play a potential therapeutic role. They showed that overexpression of miRNA‐21 has a protective function by inhibiting the PTEN signaling pathway and apoptosis to prevent hypoxia‐reperfusion (HR) and myocardial injury.^22^ This research aims to assess the diagnostic properties of exo‐miRNA‐21‐5p/3p in CAD patients to distinguish between patients and healthy subjects. To determine exo‐miRNA‐21‐5p/3p expression levels, we collected male Wistar rat serum from induced MI and control groups. This is the first study to consider exo‐miRNA‐21‐5p and exo‐miRNA‐21‐3p expression in CAD patients simultaneously, along with their biochemical factors.

## 2 MATERIALS AND METHODS

### 2.1 Human subjects

This study randomly included persons over 35 years of age: 135 patients with coronary artery disease, including subjects undergoing angiography at Ghaem Hospital, and 150 healthy participants. We classified people who underwent coronary angiography and had coronary stenosis >50% as coronary patients.^23^ Exclusion criteria for patients included: <50% stenosis, history of chronic systemic and inflammatory disease, cardiomyopathy, pregnant or lactating women, renal abnormalities, and immunosuppressive drugs such as azathioprine and tacrolimus. Also, healthy subjects were without symptoms, signs and history of angiography for coronary artery disease. In addition, Exclusion criteria for healthy group included: hypertension, vascular disease, dyslipidemia, diabetes mellitus, history of stroke and infarction, dyslipidemia and acute hypercholesterolemia family history, family history of coronary artery disease.

### 2.2 Animal samples

According to the animal table, 26 male Wistar rats (weight 250–300 g and 12 weeks old) were equally divided into induction of MI and control groups. Each group was kept in its own cage, and standard conditions such as the proper temperature (25°C), humidity, light, sufficient water, and food were produced in the animal room of the Mashhad Faculty of Medicine for both groups.

### 2.3 Ethics

Participants provided informed written consent. Also, Anonymity and confidentiality were maintained throughout the study. Furthermore, all experimental protocols were performed according to the guidelines approved by the ethics committee of Mashhad University of Medical Sciences (MUMS).

### 2.4 The laboratory characteristics and clinical baseline of all subjects

As shown in Table 1, all CAD patients and healthy group have similar ages. Patients and the control group provided information on low‐density lipoprotein cholesterol (LDL‐C), high‐density lipoprotein cholesterol (HDL‐C), body mass index (BMI), total cholesterol (TC), systolic blood pressure (SBP), diastolic blood pressure (DBP), high‐sensitivity C‐reactive protein (hs‐CRP), and smoking. Total cholesterol, hs‐CRP, LDL‐C, and HDL‐C were in the patient's record before angiography. The patient's blood pressure was monitored upon entrance to the angiography department according to World Health Organization guidelines.^24^ Cardiologists assessed the angiographic data. Positive findings (stenosis above 50%) were considered a diagnostic criterion of vascular stenosis. To eliminate observer bias, only one cardiologist examined the results in this study.

**Table 1:** Comparison of the baseline characteristics between different studied groups.

Parameters	Healthy subjects (*n* = 150)	CAD patients (*n* = 135)	*p* Value
Age (years)	53.1 ± 11.5	54.00 ± 10	0.41
Gender (male/female)	33/35	34/33	0.319
Serum HDL‐C (mg/dL)	56.15 ± 10.12	34.27 ± 7.77	<0.001*
Serum hs‐CRP (mg/L)	2.87 ± 1.68	7.83 ± 2.74	<0.001*
Serum LDL‐C (mg/dL)	93.53 ± 29	95.20 ± 31	0.428
BMI (kg/m^2^)	24.41 ± 4.7	24.83 ± 3.9	0.34
SBP (mmHg)	124.7 ± 10/4	131.8 ± 16.9	<0/001*
DBP (mmHg)	78.6 ± 6.7	79.2 ± 11.2	0.052
TC (mg/dL)	176.46 ± 41.38	177.37 ± 50.11	0.82
No Smoking	58 (81.7%)	58 (86.6%)	0.308
Yes	10 (14.1%)	9 (13.4%)	

*Note*: Data are shown as mean ± SD or median (IQR); *T*‐Student test and Mann–Whitney *U* test were used.

Abbreviations: BMI, body mass index; DBP, diastolic blood pressure; HDL‐C, high‐density lipoprotein cholesterol; hs‐CRP, high‐sensitivity C‐reactive Protein; IQR, interquartile range; LDL‐C, low‐density lipoprotein cholesterol; SBP, systolic blood pressure; SD, standard deviation; TC, total cholesterol.

* *p* Value significant level, <0.05.

### 2.5 Animal MI induction using isoprenaline hydrochloride

In this study, the induction procedure for MI using isoprenaline hydrochloride (ISO) was performed according to the methods of previous studies.^25^, ^26^ First, all animals were weighed on an electronic scale. Next, 150 mg of ISO powder (Sigma‐Aldrich) was dissolved in 2 mL of water for injection. 150 mg/kg of this solution was then injected subcutaneously for 2 consecutive days. Immediately after the last injection, exosomes were extracted from serum samples, and exo‐miRNA‐21‐5p expression levels were assessed by quantitative PCR (qPCR). We injected the healthy group with an equal volume of saline (pH: 7.2) for 2 days to equalize the injection stress between the MI rats and the control group.

### 2.6 Blood sampling

Blood samples (5 mL) from human subjects were collected in ethylenediaminetetraacetic acid (EDTA) tubes from the antecubital vein in a sitting position. In addition, for animal samples, all animals were anesthetized with ketamine hydrochloride (50 mg/kg) and xylazine hydrochloride (10 mg/kg), and 2 mL of blood was collected from the sinus membrane of rat eyes by inserting a capillary tube. Finally, blood samples were immediately centrifuged at 2800 rpm for 15 min to separate serum and frozen at −80°C for future analysis.

### 2.7 Exosome isolation

Serum samples were centrifuged at 2800 rpm for 12 min to remove sediment debris. We then obtained the serum exosomes using the exosome isolation kit (CIBBIOTECH) according to its method. Purified exosomes were stored at −80°C. The exosome size was assessed by dynamic light scattering (DLS) and transmission electron microscopy (TEM).

### 2.8 DLS

The diameter of the exosome was determined by the DLS technique with a particle size analyzer (Vasco Instruments, Cordouan Technologies). In this study, phosphate‐buffered saline (PBS) was used, and its refractive index was 1.33 with a viscosity of 0.891. We diluted 20 μL of purified exosomes in 100 μL of phosphate salt buffer (1:5). This information is needed to analyze the results.

### 2.9 TEM

For TEM analysis, 5 μL of exosome suspension was resuspended in PBS and then fixed with 1% glutaraldehyde. Ten microliters of the sample was placed on a carbon‐coated grids at room temperature to dry. The grid was washed twice with sterile PBS for 5 min and finally stained with 1% uranyl acetate for 5 min. Finally, we observed the samples under TEM.

### 2.10 RNA extraction and polyadenylation reaction

Total RNA was extracted from 1 mL of serum using the protocol‐based RNX‐PLUS kit (SINACLON) and according to the manufacturer's instructions. The concentration of RNA samples was measured by nanodrop at 260/280 nm (Thermo Scientific). Then, the polyadenylation reaction was carried out by BON‐miRNA polyadenylation kit protocol (Bonbiotech).

### 2.11 Complementary DNA (cDNA) synthesis

We synthesized the first strand of cDNA by using the BON‐miRNA cDNA synthesis kit (Bon‐Biotech), as described by the manufacturers.

### 2.12 Relative quantification PCR

The SYBR Green real‐time PCR reaction was used to evaluate the expression levels of exo‐miRNA‐21‐5p and exo‐miRNA‐21‐3p on the ABI STEP ONE real‐time PCR system (Applied Biosystems). It was accomplished by according to the BON‐miRNA QPCR kit (Bon‐Biotech) protocols. Table 2 lists the primers used in this study. Also, snRNA U6 and U87 (the housekeeping gene) was used to normalize the relative expression levels for human and animal samples, respectively.^27^

**Table 2:** Quantitative polymerase chain reaction primers.

Primer's name	Forward primers	Reverse primer (universal)
miRNA‐21‐5p	5́‐GGCTTGTCAGACGATG‐3́	5́‐GAGCAGGGTCCGAG‐3́
miRNA‐21‐3p	5́‐ACCAGACGATGGC‐3́	
U6	5́‐AAGGATGACACGCAAA‐3́	
U87	5́‐ACTTATGTTTTTGCCGTT‐3́	

## 3 ANALYSIS

All data analyses were performed using the social science statistical software package (SPSS Inc.). The Kolmogorov–Smirnov test was used to identify normally distributed variables. The Mann–Whitney *U* test was used for continuous variables if they were not normally distributed, and the Student *t* test for normally distributed variables. RT‐PCR results were analyzed using the $2^{‐∆∆C_{t}}$ method, and factor change was reported.^28^ A graph of relative expression levels plotted with GraphPad Prism version 8. Statistical significance was determined when the *p* < 0.05.

## 4 RESULTS

### 4.1 Human subjects

This study included 135 patients with coronary artery disease with a mean age of 54 ± 10 years, as well as 150 healthy subjects defined as controls with a mean age of 53.1 ± 11.5 years. 14.07% of the total population were smokers, and there was no significant difference between CAD and the control group. Clinical features, including HDL‐C, LDL‐C, TC, BMI, SBP, DBP, and hs‐CRP, were collected in Table 1. There was no significant difference (*p* > 0.05) in gender, age, LDL‐C, TC, DBP, and BMI, between coronary patients and healthy subjects. The mean HDL‐C level was significantly lower (34.27 ± 7.77 mg/dL) in the CAD group compared with the control group (56.15 ± 10.12 mg/dL). In addition, the *t*‐test showed that the hs‐CRP and SBP of the patients were significantly higher (*p* < 0.05).

### 4.2 Exo‐miRNA‐21‐5p/3p expression in CAD and control groups

**Figure 1:**
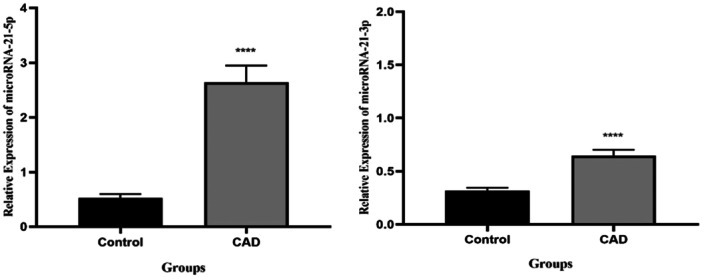
The relative expression of serum exosomal micoRNA‐21‐5p/3p in CAD and Control groups, **** (*p* < 0.0001).

First, by examining the specific melting curves of exo‐miRNA‐21‐5p/3p, genomic DNA contamination and primer function were confirmed. Then, the Kolmogorov–Smirnov test was performed to determine the regularity of the distribution of variables. Results indicated that all data from the CAD and control groups were not normally distributed (*p* < 0.05), and the Mann–Whitney *U* test was used. $2^{‐∆∆C_{t}}$ was used to assess the expression level of exo‐miRNA‐21‐5p/3p in CAD and control groups. Figure 1 shows that the relative serum exo‐miRNA‐21‐5p expression was significantly higher (fivefold) in the CAD group compared with the control group (*p* < 0.05). Similarly, the exo‐miRNA‐21‐3p expression level of the CAD group was upregulated (2.3‐fold) significantly compared with the control group (*p* < 0.05).

### 4.3 Validation of serum‐derived exosomes characterization using TEM and DLS

**Figure 2:**
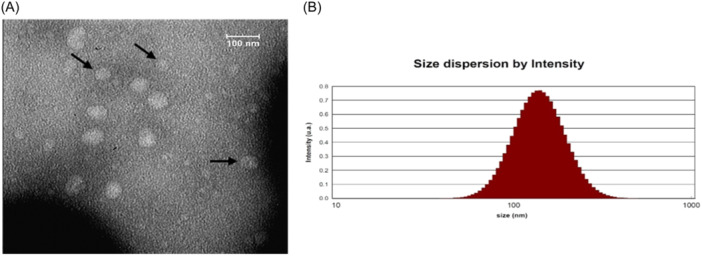
Serum‐derived exosomes characteristics. (A) Microscopic image with transmission electron microscopy, the sphericity of the exosomes can be seen in the image, black arrows showed exosomes, scale bar = 100 nm. (B) Size distribution of exosomes by dynamic light scattering.

As shown in Figure 2, the structure and size distribution of serum‐derived exosomes were determined by TEM and DLS techniques. TEM demonstrated that (Figure 2A) the integrity of the exosomes' membrane and spherical morphology were preserved during the separation process and the spherical structure was not disassembled. Also, DLS measurements indicated the diameter of the serum‐derived exosomes was approximately 100 nm (Figure 2B). According to the accuracy of the above results, isolated exosomes could be used in further studies.

### 4.4 The diagnostic value of serum exo‐miRNA‐21‐5p/3p

**Figure 3:**
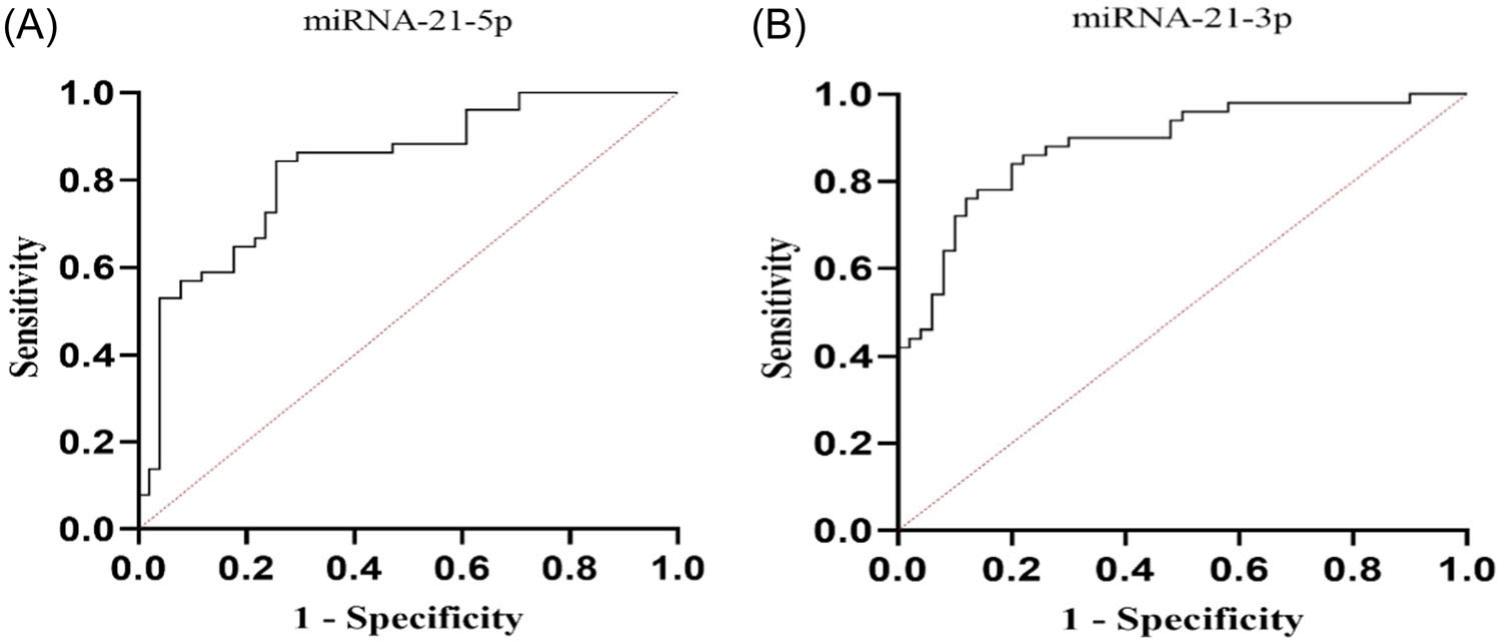
Receiver‐operating‐characteristic curve of miRNA‐21, (A) Diagnostic potential of miRNA‐21‐5p, AUC: 0.8293 (0.7496–0.9090 at 95% confidence interval [CI]) with 70% specificity and 86% sensitivity, (B) Diagnostic potential of miRNA‐21‐3p, 0.8840 (0.8186–0.9494 at 95% CI) with 80% specificity and 84% sensitivity.

We evaluated the area under the curve (AUC) of the receiver‐operating‐characteristic (ROC) diagram to analyze the cyclic exo‐miRNA‐21‐5p/3p as a potential biomarker of CAD. AUC > 80% is an acceptable value for predicting coronary artery disease. According to the curve analysis (ROC) in Figure 3, the AUC scores of exo‐miRNA‐21‐5p and exo‐miRNA‐21‐3p were 0.8293 (0.7496–0.9090 at 95% CI) and 0.8840 (0.8186–0.9494 at 95% CI), respectively. This demonstrates that exo‐miRNA‐21‐5p/3p show significant predictive power (81%) to distinguish CAD patients from healthy participants.

### 4.5 Interaction of serum exo‐miRNA‐21‐5p/3p with risk factors in CAD subjects

Pearson's correlation analysis was used to evaluate positive or negative correlations between exo‐miRNA‐21 and risk factors. Exo‐miRNA‐21‐3p was significantly positively correlated with LDL‐C and hs‐CRP (*p* < 0.05). However, exo‐miRNA‐21‐5p was negatively correlated with LDL‐C and hs‐CRP, but not significantly (*p* = 0.8). Although HDL‐C was negatively correlated with exo‐miRNA‐21‐3p, it was positively correlated with exo‐miRNA‐21‐5p, which was not statistically significant (*p* > 0.05).

### 4.6 Animal samples

**Figure 4:**
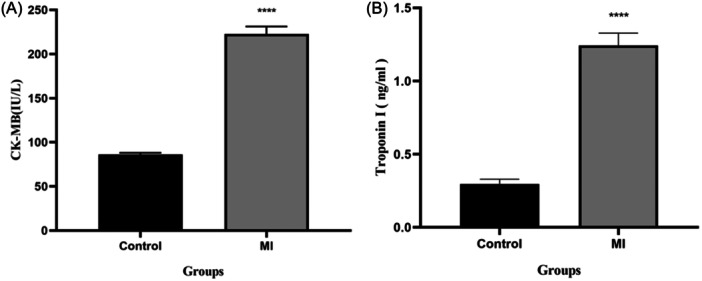
Comparison of creatine kinase‐MB (A) and Troponin I (B) concentrations in control group and acute myocardial infarction. IU, international unit, ***p < 0.0001.

ISO is a catecholamine and causes severe myocardial necrosis.^29^, ^30^ The results showed in Figure 4 an increase in CK‐MB and troponin I after ISO‐induced stroke in the MI group compared with the control group. The mean serum CK‐MB activity in the control and MI groups was 86 ± 1 and 222 ± 8 IU/L, respectively, indicating a significant difference (*p* < 0.0001). Furthermore, ROC curve analysis indicated that elevated CK‐MB levels (>144.9 IU/L) could identify the occurrence of stroke with 100% specificity and sensitivity. The mean troponin I concentration in the control group was 0.29 ng/mL, indicating a significant difference (*p* < 0.0001) compared with the MI group of 1.24 ng/mL. The ROC curve results can be used to diagnose stroke for troponin I at concentrations greater than 0.68 ng/mL with 100% specificity and sensitivity in rats.

### 4.7 Histopathological alteration in myocardium

**Figure 5:**
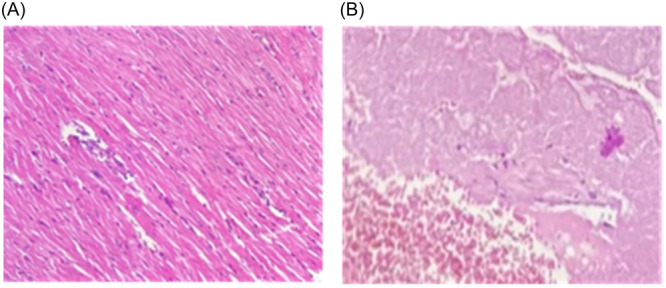
(A) Histopathological normal myocardial tissue with longitudinal connections and no necrosis and infiltration of inflammatory cells in control group. (B) The rupture of muscle fibers with loss of transverse striations in myocardial infarction group.

MI causes cardiomyocyte cell damage and cell necrosis by leakage of lysosomal enzymes from damaged cells into the cytoplasm.^31^ The results of the necrosis study by light microscopy and using hematoxylin and eosin staining groups are as follows in the control group and MI (Figure 5). The myocardial tissue in the control group was observed to be fascicles of muscle with longitudinal connections and without necrosis or inflammatory cell infiltration. In the MI group, superficial fibroid necrosis was observed along with transverse striations, muscle fiber rupture, inflammatory cell infiltration, and bleeding.

### 4.8 Expression of exo‐miRNA‐21‐5p in MI and control groups

**Figure 6:**
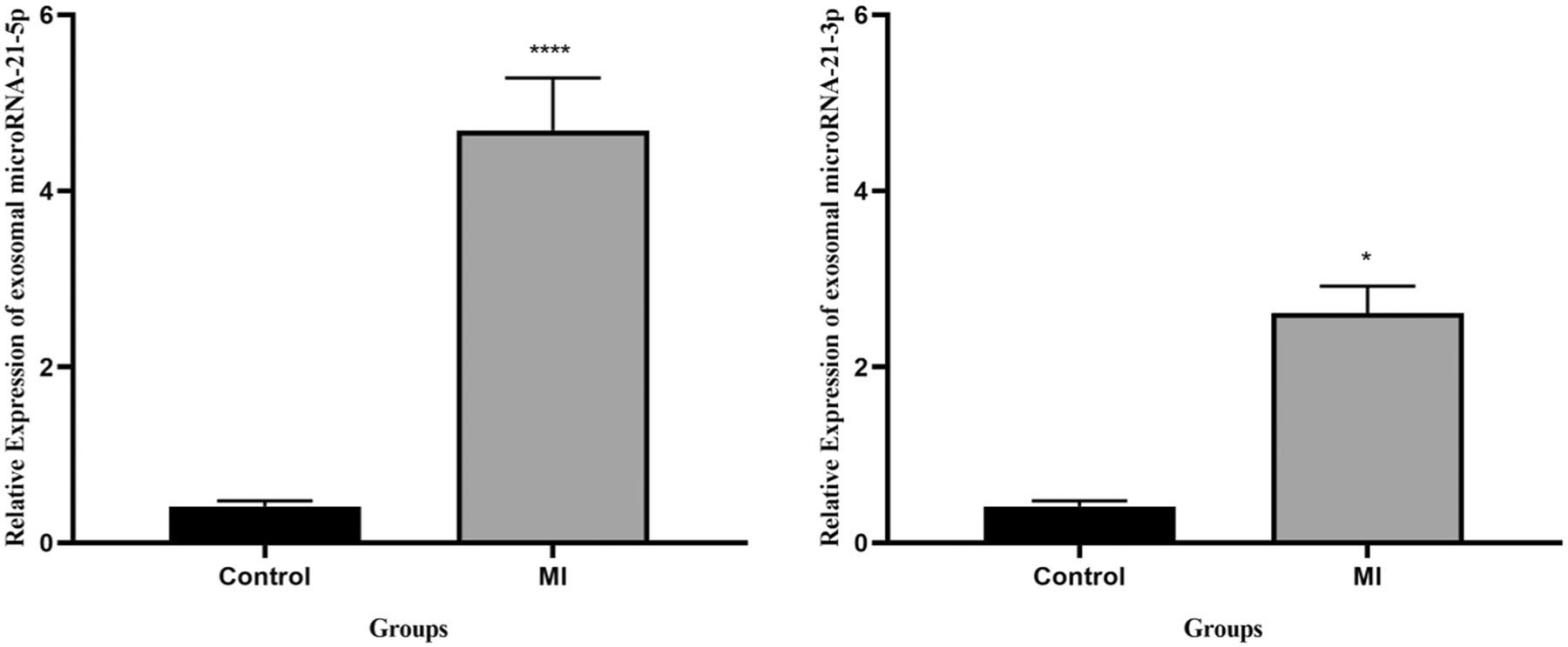
The relative expression levels of serum exosomal micoRNA‐21‐5p/3p.

The D'Agostino–Pearson, Shapiro–Wilk, and Kolmogorov–Smirnov normality tests showed that all data from both control and MI were normally distributed (*p* > 0.05). Next, we evaluated the expression levels of serum exo‐miRNA‐21‐5p/3p in the MI and control groups (Figure 6). Also, the average fold changes in the exo‐miRNA‐21‐5p and exo‐miRNA‐21‐3p genes in the MI group compared with the control group were 11.4‐ and 6.38‐fold (*p* < 0.05).

### 4.9 The diagnostic value of serum exo‐miRNA‐21‐5p/3p

**Figure 7:**
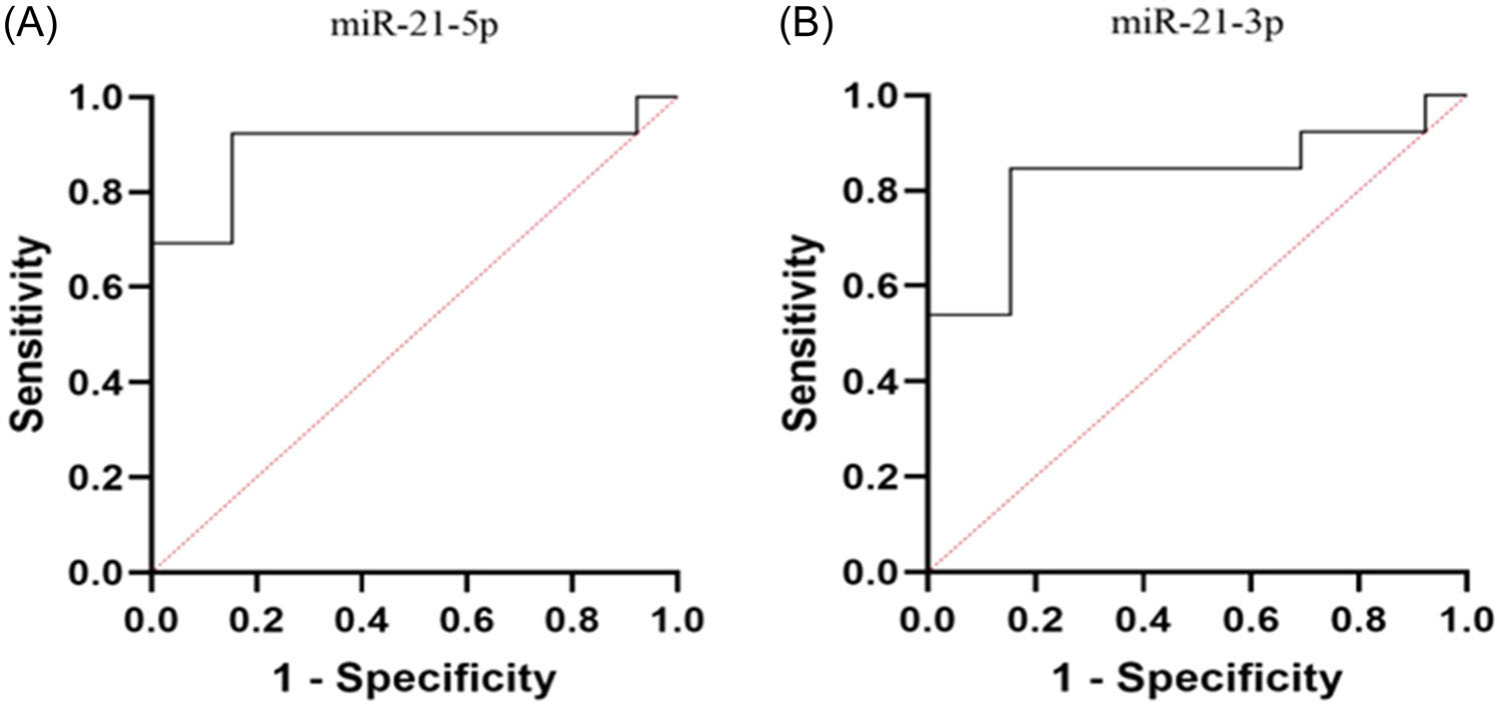
Receiver‐operating‐characteristic curve of miRNA‐21, (A) Diagnostic potential of miRNA‐21‐5p, area under the curve: 0.92 (0.79–1 at 95% confidence interval [CI]) with 100% specificity and 92.3% sensitivity, (B) Diagnostic potential of miRNA‐21‐3p, 0.82 (0.65–1 at 95% CI) with 85% specificity and 84% sensitivity.

ROC curve analysis determined the diagnostic value of exo‐miRNA‐21‐5p/3p using the AUC score. According to Figure 7, the AUC of exo‐miRNA‐21‐5p and exo‐miRNA‐21‐3p were 0.92 (95% CI, 0.79–1, *p* = 0.0002) and 0.82 (95% CI, 0.65–1, *p* = 0.004) and a cutoff value equal to or greater than 1.369 predicted MI status with 92.3% sensitivity and 100% specificity for exo‐miRNA‐21‐5p and equal to or greater than 1.27 with 84% sensitivity and 85% specificity for exo‐miRNA‐21‐3p. Based on the above results, exo‐miRNA‐21‐5p/3p may be a novel prognostic biomarker for MI in rats.

## 5 DISCUSSION

CAD is one of the main causes of morbidity and mortality worldwide.^2^ Serum troponin and CK‐MB levels are the key MI diagnostic tests. Although, cardiac troponin is used to diagnose MI because of its high sensitivity and specificity; however, its blood level increases only 3–4 h after cardiac injury. According to several reports, the presence of some interfering factors, such as rheumatoid factor and fibrin clots, leads to a false positive assay in troponin levels.^32^ These biomarkers cannot be used in early detection^33^; thus, appropriate diagnostic methods are needed to more effectively treat and reduce mortality.^34^ Many researchers have studied miRNA in molecular and cellular functions, and disease processes in recent years.^35^, ^36^ MiRNAs, also known as noncoding RNAs, play critical roles in apoptosis, cell proliferation, and angiogenesis.^37^, ^38^ Cell damage and disease release a large number of miRNAs into the blood quickly. Therefore, they are valuable candidates for prognosis and diagnosis in many chronic diseases, including CAD.^39^ Due to their small size and linking with carriers such as extracellular vesicles (EVs), circulating miRNAs are resistant to RNase activity. This stability makes them suitable for CAD diagnostic marker.^39^ Recently, miRNA‐21 has been introduced as a potential biomarker for CAD and AMI diagnosis.^40^ Wang et al. showed miRNA‐21 could be involved directly and indirectly in the pathophysiological processes of atherosclerosis through apoptosis inhibition.^41^, ^42^ Dong Yiheng discovered that miRNA‐21 was upregulated in cardiac graft fibrosis. Also, this miRNA is overexpressed in patients with rejection of heart transplantation.^43^ Li Qiao and colleagues reported in 2019 that exosomes containing miRNA‐21‐5p help with heart repair by increasing angiogenesis and enhancing the expression level of vascular endothelial growth factor (VEGF) in patients with heart failure. Also, they showed exo‐miRNA‐21‐5p is a mediator of the Akt pathway with the aim of cardiomyocyte survival.^44^ Furthermore, Zhang and coworkers illustrated in their research on rats with AMI that upregulation of miRNA‐21 could improve cardiac function by reducing myocardial cell apoptosis.^45^ In agreement with Zhang JC's research, Liao showed exo‐miRNA‐21 had an antiapoptotic function and prevented the apoptosis of cardiomyocytes, causing the proliferation of endothelial cells by downregulating programmed cell death 4 (PDCD4).^46^ In this study, we compared the relationship between various demographic and biochemical parameters such as age, gender, BMI, LDL‐C, hs‐CRP, and alters in serum exosomal miRNA‐21‐5p/3p expression in patients with CAD in comparison with healthy subjects. We also evaluated the expression level of serum exo‐miRNA‐21‐5p/3p in samples from rats that induced MI, as well as in the control group. Our statistical analysis of the data indicates that exo‐miRNA‐21‐5p/3p has a potential role in the diagnosis of coronary artery disease. According to the results, risk factors like HDL‐C, hs‐CRP, and SBP are significantly related to high expression of exo‐miRNA‐21‐5p/3p in the CAD group. Additionally, the association between smoking and exo‐miRNA‐21‐5p/3p expression is considered a risk factor, but its impact is not significant. Some studies by Guo‐fu Zhu and others in 2019 showed miRNAs related to inflammation (such as miRNA‐21) are highly expressed, which is obviously positively correlated with the increase in the levels of inflammatory markers such as hs‐CRP in coronary artery stenosis.^47^ Similarly, other research on patients with stable CAD has shown that elevating serum miRNA‐21 may contribute to the development of inflammatory pathways in the pathogenesis of CAD or may be related to the effects of inflammatory diseases on atherosclerosis and acute coronary syndrome.^48^ In line with previous reviews, the present study indicates exo‐miRNA‐21‐3p has a significant positive correlation with hs‐CRP levels in CAD patients. Compared with exo‐miRNA‐21‐3p, exo‐miRNA‐21‐5p has a negative correlation with hs‐CRP levels, but this relationship is not significant. Hence, a significant increase in the expression level of exo‐miRNA‐21‐5p/3p may be in response to the inflammatory process. Finally, the mechanisms of signaling pathways are not yet well understood, and further studies on the role of exo‐miRNA‐21 in coronary plaques are needed. A study by Dawid Miśkowiec revealed that the expression levels of miRNA‐21 had an inverse correlation with the lipid profile, including HDL‐C, in patients with coronary disease. According to their research, the upregulation of miRNA‐21 due to lipid profile may be related to less plaque formation stimulation.^49^ Our findings are indirectly related to previous studies, which showed that exo‐miRNA‐21 causes a decrease in atherogenic status. The predictive power of exo‐miRNA‐21‐5p/3p was evaluated based on the area under the curve analysis. The diagnostic values of exo‐miRNA‐21‐5p and exo‐miRNA‐21‐3p are 82% and 88%, respectively, with impressive specificity and sensitivity. Similar results were obtained from Dinesh Kumar et al.^50^ Therefore, this study presents for the first time a clinical report on exo‐miRNA‐21‐5p/3p in serum, which is an important noninvasive and inexpensive biomarker for predicting and diagnosing the state of CAD. Also, for the first time, the results of our investigation on animal‐induced MI samples showed significant overexpression (*p* < 0.05) of exo‐miRNA‐21‐5p/3p in serum samples of MI rats compared with the control group. This increasing level may promote a key pathway to reduce apoptosis and preserve cardiomyocyte cells. In confirmation of our hypothesis, Nana He et al. stated that exo‐miRNA‐21‐5p could play a protective role for myocardial cells against apoptosis and could be used as a novel therapy for cardiovascular disease.^51^ Also, Ken Watanabe showed upregulation of the level of miRNA‐21 expression occurs after angiotensin II (Ang II) infusion in mice, which reduces the expression level of PDCD4 compared to the control group.^52^ Accordingly, our reviews, along with other studies, suggest elevating the serum level of exo‐miRNA‐21‐5p may be effective for the diagnosis of AMI, and this study considers this issue through ROC curve analysis. As a result, in rats, exo‐miRNA‐21‐5p can predict AMI status with 92.3% sensitivity and 100% specificity. Similar to our results, Feng Wang, based on the results of the ROC analysis in the cohort study, revealed that miRNA‐21‐5p could be a powerful diagnostic biomarker in AMI patients.^53^ In addition, Han Ding and colleagues illustrated in 2020 that miRNA‐21‐5p could be considered a clinically rapid diagnostic and prognostic biomarker with 89.7% sensitivity and 82.8% specificity for heart failure.^54^ At the end, despite considering most research information, our research has limitations: First, although the estimated population is small, the results seem to be consistent. Second, we have organized limited regional studies, and future investigations need to be conducted in multiple centers. Third, the lack of follow‐up in the human models prevents us from exploring the possible role of exo‐miRNA‐21‐5p/3p in CAD prognosis. Eventually, more patients from the general cardiac population will need to be screened for further study to clarify the relationship observed at the human molecular level.

## 6 CONCLUSION

Exo‐miRNA‐21‐3p overexpression positively correlates with hs‐CRP and LDL‐C levels in patients with coronary artery disease, while it negatively correlates with HDL‐C. The correlation between exo‐miRNA‐21‐5p and biochemical factors (HDL‐C, LDL‐C, and hs‐CRP) is opposite to that between exo‐miRNA‐21‐3p, but the results are not significant. Obviously, exo‐miRNA‐21‐5p/3p play a key role in the lipid profile. It is necessary to determine the role of exo‐miRNA‐21 as a target for coronary artery disease diagnosis and treatment. Also, our study examined changes in the expression level of serum‐derived exo‐miRNA‐21‐5p after induction of MI in an animal model, and we confirmed that the expression of exo‐miRNA‐21‐5p/3p increased in the MI group compared to the control.

## AUTHOR CONTRIBUTIONS

**Reza Sahebi**: Writing—original draft; investigation; methodology; project administration; supervision; validation; conceptualization; funding acquisition; visualization. **Fatemeh Gandomi**: Writing—review and editing; data curation. **Mitra shojaei**: Writing—review and editing. **Effat Farrokhi**: Formal analysis; software; data curation; resources.

## CONFLICT OF INTEREST STATEMENT

The authors declare no conflict of interest.

## ETHICS STATEMENT

The study was reviewed and approved by the Human Research Ethics Committee of Mashhad and Shahrekord Universities of Medical Sciences in 2018.

## TRANSPARENCY STATEMENT

The lead author Reza Sahebi, Effat Farrokhi affirms that this manuscript is an honest, accurate, and transparent account of the study being reported; that no important aspects of the study have been omitted; and that any discrepancies from the study as planned (and, if relevant, registered) have been explained.

## Data Availability

The data that support the findings of this study are available on request from the corresponding author. The data are not publicly available due to privacy or ethical restrictions.

## References

[1] Malakar AK , Choudhury D , Halder B , Paul P , Uddin A , Chakraborty S . A review on coronary artery disease, its risk factors, and therapeutics. J Cell Physiol. 2019;234(10):16812‐16823. 30790284 10.1002/jcp.28350

[2] Mastoi QU , Wah TY , Gopal Raj R , Iqbal U . Automated diagnosis of coronary artery disease: a review and workflow. Cardiol Res Pract. 2018;2018:2016282. 29507812 10.1155/2018/2016282 PMC5817359

[3] Garavand A , Salehnasab C , Behmanesh A , Aslani N , Zadeh AH , Ghaderzadeh M . Efficient model for coronary artery disease diagnosis: a comparative study of several machine learning algorithms. J Healthc Eng. 2022;2022(1):5359540. 36304749 10.1155/2022/5359540 PMC9596250

[4] Casavecchia G , Galderisi M , Novo G , et al. Early diagnosis, clinical management, and follow‐up of cardiovascular events with ponatinib. Heart Fail Rev. 2020;25:447‐456. 32026180 10.1007/s10741-020-09926-y

[5] Ghobrial M , Haley HA , Gosling R , et al. The new role of diagnostic angiography in coronary physiological assessment. Heart. 2021;107:783‐789. 33419878 10.1136/heartjnl-2020-318289 PMC8077221

[6] Randazzo MJ , Elias P , Poterucha TJ , et al. Impact of cardiac size on diagnostic performance of single‐photon emission computed tomography myocardial perfusion imaging: insights from the REgistry of Fast Myocardial Perfusion Imaging with NExt generation single‐photon emission computed tomography. Eur Heart J Cardiovasc Imaging. 2024;25:jeae055. 10.1093/ehjci/jeae055 PMC11210974 38445511

[7] Rezaei M , Zahmatkeshan N . Effect of irregular imaging on the quality of myocardial perfusion myocardial single‐photon emission computed tomography images. Med Clín Práctica. 2023;6(1):100353.

[8] Ling H , Guo Z , Shi Y , Zhang L , Song C . Serum exosomal microRNA‐21, microRNA‐126, and PTEN are novel biomarkers for diagnosis of acute coronary syndrome. Front Physiol. 2020;11:654. 32595526 10.3389/fphys.2020.00654 PMC7300246

[9] Kura B , Kalocayova B , Devaux Y , Bartekova M . Potential clinical implications of miR‐1 and miR‐21 in heart disease and cardioprotection. Int J Mol Sci. 2020;21(3):700. 31973111 10.3390/ijms21030700 PMC7037063

[10] Wang M , Yu F , Ding H , Wang Y , Li P , Wang K . Emerging function and clinical values of exosomal MicroRNAs in cancer. Mol Ther Nucleic acids. 2019;16:791‐804. 31163321 10.1016/j.omtn.2019.04.027 PMC6545365

[11] Jakubik D , Fitas A , Eyileten C , et al. MicroRNAs and long non‐coding RNAs in the pathophysiological processes of diabetic cardiomyopathy: emerging biomarkers and potential therapeutics. Cardiovasc Diabetol. 2021;20:55. 33639953 10.1186/s12933-021-01245-2 PMC7916283

[12] Collins L , Binder P , Chen H , Wang X . Regulation of long non‐coding RNAs and microRNAs in heart disease: insight into mechanisms and therapeutic approaches. Front Physiol. 2020;11:543573. 10.3389/fphys.2020.00798 PMC7365882 32754048

[13] Zheng D , Huo M , Li B , et al. The role of exosomes and exosomal microRNA in cardiovascular disease. Front Cell Dev Biol. 2020;8:616161. 33511124 10.3389/fcell.2020.616161 PMC7835482

[14] Bellin G , Gardin C , Ferroni L , et al. Exosome in cardiovascular diseases: a complex world full of hope. Cells. 2019;8(2):166. 30781555 10.3390/cells8020166 PMC6406975

[15] Geng T , Song Z‐Y , Xing J‐X , Wang B‐X , Dai S‐P , Xu Z‐S . Exosome derived from coronary serum of patients with myocardial infarction promotes angiogenesis through the miRNA‐143/IGF‐IR pathway. Int J Nanomed. 2020;15:2647. 10.2147/IJN.S242908 PMC7183550 32368046

[16] Ruan Y , Guo Y , Zheng Y , et al. Cardiovascular disease (CVD) and associated risk factors among older adults in six low‐and middle‐income countries: results from SAGE Wave 1. BMC Public Health. 2018;18(1):778. 29925336 10.1186/s12889-018-5653-9 PMC6011508

[17] Zhou S , Jin J , Wang J , et al. miRNAS in cardiovascular diseases: potential biomarkers, therapeutic targets and challenges. Acta Pharmacol Sin. 2018;39(7):1073‐1084. 29877320 10.1038/aps.2018.30 PMC6289363

[18] Kaur A , Mackin ST , Schlosser K , et al. Systematic review of microRNA biomarkers in acute coronary syndrome and stable coronary artery disease. Cardiovasc Res. 2020;116(6):1113‐1124. 31782762 10.1093/cvr/cvz302

[19] Dai B , Wang F , Nie X , et al. The cell type–specific functions of miR‐21 in cardiovascular diseases. Front Genet. 2020;11:563166. 33329700 10.3389/fgene.2020.563166 PMC7714932

[20] Yan M , Chen C , Gong W , et al. miR‐21‐3p regulates cardiac hypertrophic response by targeting histone deacetylase‐8. Cardiovasc Res. 2015;105(3):340‐352. 25504627 10.1093/cvr/cvu254

[21] Li X , Wei Y , Wang Z . microRNA‐21 and hypertension. Hypertension Res. 2018;41(9):649‐661. 10.1038/s41440-018-0071-z 29973661

[22] Zhou S , Sun Y , Zhao K , et al miR‑21/PTEN pathway mediates the cardioprotection of geniposide against oxidized low‑density lipoprotein‑induced endothelial injury via suppressing oxidative stress and inflammatory response. Int J Mol Med. 2020;45(5):1305‐1316. 32323738 10.3892/ijmm.2020.4520 PMC7138279

[23] Kishore AHN , Jayanthi V . Automatic stenosis grading system for diagnosing coronary artery disease using coronary angiogram. Int J Biomed Eng Technol. 2019;31(3):260‐277.

[24] World Health Organization . *Guideline for the Pharmacological Treatment of Hypertension in Adults*. World Health Organization; 2020. 34495610

[25] Ferreira N , Sousa R , Carvalho E , Lobo P . Experimental model of myocardial infarction induced by isoproterenol in rats. Braz J Cardiovasc Surg. 2011;26(3):469‐476. 10.5935/1678-9741.20110024 22086586

[26] Boarescu P‐M , Boarescu I , BOCȘAN 4, IC , et al. Experimental model of acute myocardial infarction for evaluation of prevention and rehabilitation strategies in cardiovascular diseases—a pilot study. Balneo Res J. 2019;10:288‐293.

[27] Kmecova Z , Veteskova J , Lelkova‐Zirova K , et al. Disease severity–related alterations of cardiac microRNAs in experimental pulmonary hypertension. J Cell Mol Med. 2020;24(12):6943‐6951. 32395887 10.1111/jcmm.15352 PMC7299706

[28] Schmittgen TD , Livak KJ . Analyzing real‐time PCR data by the comparative C T method. Nat Protoc. 2008;3(6):1101‐1108. 18546601 10.1038/nprot.2008.73

[29] Wu H , Su H , Zhu C , Wu S , Cui S , Zhou M . Establishment and effect evaluation of a stress cardiomyopathy mouse model induced by different doses of isoprenaline. Exp Ther Med. 2023;25(4):166. 36936708 10.3892/etm.2023.11865 PMC10015318

[30] Boarescu P‐M , Boarescu I , Bocșan IC , et al. Curcumin nanoparticles protect against isoproterenol induced myocardial infarction by alleviating myocardial tissue oxidative stress, electrocardiogram, and biological changes. Molecules. 2019;24(15):2802. 31374848 10.3390/molecules24152802 PMC6696485

[31] Zhang C , Gan X , Liang R , Jian J . Exosomes derived from epigallocatechin gallate‐treated cardiomyocytes attenuated acute myocardial infarction by modulating MicroRNA‐30a. Front Pharmacol. 2020;11:126. 32161548 10.3389/fphar.2020.00126 PMC7054242

[32] Chaulin AM . Some common causes of false positive increases in serum levels of cardiac troponins. Curr Cardiol Rev. 2022;18(6):e130422203527. 35422225 10.2174/1573403X18666220413124038 PMC9893154

[33] Kaier TE , Alaour B , Marber M . Cardiac troponin and defining myocardial infarction. Cardiovasc Res. 2021;117:2203‐2215. 33458742 10.1093/cvr/cvaa331 PMC8404461

[34] Mir R , Elfaki I , Khullar N , et al. Role of selected miRNAs as diagnostic and prognostic biomarkers in cardiovascular diseases, including coronary artery disease, myocardial infarction and atherosclerosis. J Cardiovasc Dev Dis. 2021;8(2):22. 33669699 10.3390/jcdd8020022 PMC7923109

[35] Çakmak HA , Demir M . MicroRNA and cardiovascular diseases. Balkan Med J. 2020;37(2):60. 32018347 10.4274/balkanmedj.galenos.2020.2020.1.94 PMC7094181

[36] Condrat CE , Thompson DC , Barbu MG , et al. miRNAs as biomarkers in disease: latest findings regarding their role in diagnosis and prognosis. Cells. 2020;9(2):276. 31979244 10.3390/cells9020276 PMC7072450

[37] Zhao Z , Sun W , Guo Z , Zhang J , Yu H , Liu B . Mechanisms of lncRNA/microRNA interactions in angiogenesis. Life Sci. 2020;254:116900. 31786194 10.1016/j.lfs.2019.116900

[38] Heydari E , Alishahi M , Ghaedrahmati F , Winlow W , Khoshnam SE , Anbiyaiee A . The role of non‐coding RNAs in neuroprotection and angiogenesis following ischemic stroke. Metab Brain Dis. 2020;35:31‐43. 31446548 10.1007/s11011-019-00485-2

[39] Zhang X , Cai H , Zhu M , Qian Y , Lin S , Li X . Circulating microRNAs as biomarkers for severe coronary artery disease. Medicine. 2020;99(17):e19971. 32332683 10.1097/MD.0000000000019971 PMC7220532

[40] Surina , Fontanella RA , Scisciola L , Marfella R , Paolisso G , Barbieri M . miR‐21 in human cardiomyopathies. Front Cardiovasc Med. 2021;8:767064. 34778418 10.3389/fcvm.2021.767064 PMC8578278

[41] Wang Z , Wang Z , Wang T , Yuan J , Wang X , Zhang Z . Inhibition of miR‐34a‐5p protected myocardial ischemia reperfusion injury‐induced apoptosis and reactive oxygen species accumulation through regulation of Notch Receptor 1 signaling. Rev Cardiovasc Med. 2019;20(3):187‐197. 31601093 10.31083/j.rcm.2019.03.545

[42] Wang W , Zheng H . Myocardial infarction: the protective role of MiRNAs in myocardium pathology. Front Cardiovasc Med. 2021;8:631817. 33748196 10.3389/fcvm.2021.631817 PMC7973051

[43] Dong Y , Peng N , Dong L , Tan S , Zhang X . Non‐coding RNAs: important participants in cardiac fibrosis. Front Cardiovasc Med. 2022;9:937995. 35966549 10.3389/fcvm.2022.937995 PMC9365961

[44] Qiao L , Hu S , Liu S , et al. microRNA‐21‐5p dysregulation in exosomes derived from heart failure patients impairs regenerative potential. J Clin Invest. 2019;129(6):2237‐2250. 31033484 10.1172/JCI123135 PMC6546482

[45] Zhang JC , Xia L , Jiang Y , et al. Effect of lncRNA GAS5 on rats with acute myocardial infarction through regulating miR‐21. Eur Rev Med Pharmacol Sci. 2019;23(19):8573‐8579. 31646590 10.26355/eurrev_201910_19173

[46] Liao Z , Chen Y , Duan C , et al. Cardiac telocytes inhibit cardiac microvascular endothelial cell apoptosis through exosomal miRNA‐21‐5p‐targeted cdip1 silencing to improve angiogenesis following myocardial infarction. Theranostics. 2021;11(1):268‐291. 33391474 10.7150/thno.47021 PMC7681094

[47] Zhu G , Chu T , Ruan Z , et al. Inflammation‐related microRNAs are associated with plaque stability calculated by IVUS in coronary heart disease patients. J Interv Cardiol. 2019;2019:1‐10. 10.1155/2019/9723129 PMC6915018 31866771

[48] Torres‐Paz YE , Gamboa R , Fuentevilla‐Álvarez G , et al. Overexpression of microRNA‐21‐5p and microRNA‐221‐5p in monocytes increases the risk of developing coronary artery disease. Int J Mol Sci. 2023;24(10):8641. 37239987 10.3390/ijms24108641 PMC10218481

[49] Miśkowiec D , Lipiec P , Wierzbowska‐Drabik K , et al. Association between microRNA‐21 concentration and lipid profile in patients with acute coronary syndrome without persistent ST‐segment elevation. Pol Arch Med Wewn. 2016;126(1‐2):48‐57. 26842377 10.20452/pamw.3267

[50] Kumar D , Narang R , Sreenivas V , et al. Circulatory miR‐133b and miR‐21 as novel biomarkers in early prediction and diagnosis of coronary artery disease. Genes. 2020;11(2):164. 32033332 10.3390/genes11020164 PMC7073535

[51] He N , Zhang Y , Zhang S , Wang D , Ye H . Exosomes: cell‐free therapy for cardiovascular diseases. J Cardiovasc Transl Res. 2020;13:713‐721. 32333198 10.1007/s12265-020-09966-7

[52] Watanabe K , Narumi T , Watanabe T , et al. The association between microRNA‐21 and hypertension‐induced cardiac remodeling. PLoS One. 2020;15(2):e0226053. 32040481 10.1371/journal.pone.0226053 PMC7010249

[53] Xu L , Tian L , Yan Z , Wang J , Xue T , Sun Q . Diagnostic and prognostic value of miR‐486‐5p, miR‐451a, miR‐21‐5p and monocyte to high‐density lipoprotein cholesterol ratio in patients with acute myocardial infarction. Heart Vessels. 2023;38(3):318‐331. 36214846 10.1007/s00380-022-02172-2 PMC9898386

[54] Ding H , Wang Y , Hu L , et al. Combined detection of miR‐21‐5p, miR‐30a‐3p, miR‐30a‐5p, miR‐155‐5p, miR‐216a and miR‐217 for screening of early heart failure diseases. Biosci Rep. 2020;40(3):BSR20191653. 32124924 10.1042/BSR20191653 PMC7080642

